# Prognostic nutritional index is a predictor of adverse outcomes in hospitalized COVID-19 patients: a single-center, retrospective cohort study

**DOI:** 10.1186/s12879-025-12244-z

**Published:** 2025-12-01

**Authors:** Dong Wu, Jiahua Li, Yunan Wang, Bingyu Long, Bangxiao Huang, Gege Liu, Juan Zhang, Yujuan Chen, Youping Qiao, Min Chen, Dongming Li, Bin Wu, Dan Huang, Yuanli Zhang, Xuanna Zhao

**Affiliations:** 1 Department of Respiratory and Critical Care Medicine, Affiliated Hospital of Guangdong Medical University, No. 57, South of Renmin Road, Zhanjiang, Guangdong 524013 China; 2 Department of Critical Care Medicine, Affiliated Hospital of Guangdong Medical University, Zhanjiang, 524013 China

**Keywords:** Prognostic nutritional index, COVID-19, Adverse prognostic outcomes, Nutrition

## Abstract

**Background:**

Nutritional status is associated with clinical outcomes in COVID-19 patients. The aim of this study was to investigate the relationship between the prognostic nutritional index (PNI) and adverse prognosis outcomes in COVID-19 patients.

**Methods:**

In this study, 1190 patients with COVID-19 who were hospitalized at the Affiliated Hospital of Guangdong Medical University from December 2022 to March 2023 were selected. Multivariate logistic regression analysis model was employed to assess the relationship between the PNI and prognosis outcomes. Restricted cubic spline (RCS) curves were used to evaluate the risk ratio of adverse prognosis outcomes with continuous PNI. The predictive accuracy of these risk factors was assessed by Receiver Operating Characteristic (ROC) curves. Finally, subgroup analysis was used to evaluate the robustness of the test results.

**Results:**

This study encompassed a cohort of 1,117 subjects. Stratification by PNI quartile revealed an increased incidence of negative prognostic indicators in the lowest quartile (Q1: 174 [62.1%] versus Q2: 119 [43%] versus Q3: 78 [27.9%] versus Q4: 44 [15.7%], *P* < 0.001). Multivariate logistic regression analysis, with adjustment for potential confounders, demonstrated that the PNI was a significant independent predictor of negative prognostic outcomes (odds ratio [OR] = 0.95, 95% confidence interval [95% CI]: 0.93–0.97, *P* < 0.001). The RCS curve demonstrated this relationship, with the PNI below 40 associated with an increased risk of adverse prognostic outcomes. The area under the ROC curve (AUC) values were 0.722 (95%CI: 0.691–0.752) for PNI, with a cutoff of 39.3, yielding a sensitivity of 0.671 and a specificity of 0.661.

**Conclusions:**

This study reveals that PNI is an independent predictor of adverse prognosis outcomes in COVID-19 patients. A higher PNI may be associated with a lower risk of COVID-19 disease progression.

**Clinical trial number:**

Not applicable.

**Supplementary Information:**

The online version contains supplementary material available at 10.1186/s12879-025-12244-z.

## Introduction

COVID-19, also known as coronavirus disease 2019, is an infectious disease caused by a coronavirus called SARS-CoV-2 [1]. As of November 2023, there have been over 750 million confirmed cases and more than 6.9 million deaths worldwide [2]. The pandemic continues to spread rapidly in many areas, resulting in more infections and fatalities. Therefore, the global effort to combat COVID-19 remains paramount and cannot be overlooked.

Malnutrition is a state in which the body’s protein intake is insufficient due to factors such as disease, starvation, and age, leading to changes in body composition and ultimately impairing physical and mental functions [3]. Malnutrition can adversely affect the function of the immune system and impact disease outcomes [3]. Malnutrition not only undermines the efficacy of the immune apparatus and exacerbate the severity of viral infections, but also amplifies the peril of ensuing complications [4, 5]. Concurrently, it is prevalent that contraction of COVID-19 engenders manifestations such as impaired appetite and smell, resulting in reduced food intake and malnutrition [6, 7]. There is a bidirectional relationship between malnutrition and the novel coronavirus [8]. Therefore, it is crucial to maintain the optimal nutritional status for enhancing immune function, improving the prognosis of patients with viral infections, and mitigating the severity of the disease.

Several indicators are used to evaluate the nutritional status of the human body, such as body mass index (BMI), waist circumference, body fat content, and the prognostic nutritional index (PNI) [9]. The PNI is a composite index that combines the lymphocyte (LY) count and serum albumin (ALB) level to assess a patient’s nutritional status and immune function [10, 11]. It is widely used to evaluate patients’ nutritional status and predict the risk of developing diseases in clinical practice [12, 13]. Lower PNI values have been associated with disease severity and mortality in COVID-19 patients according to previous research [14, 15]. A meta-analysis by Hung et al. involving 13 studies with over 4,200 patients showed that individuals who experienced adverse outcomes exhibited significantly lower PNI levels on average, and the odds of mortality decreased by approximately 16% for each unit increase in PNI [16]. In the study by Mathioudakis et al. in Athens, Greece, it was confirmed that PNI can also predict outcomes in COVID-19 patients during the Omicron wave [17]. However, during the Omicron wave, there is a significant lack of large-cohort studies conducted in Asian populations investigating the impact of PNI on multiple adverse prognostic outcomes, such as death, intubation/mechanical ventilation, or admission to the intensive care unit (ICU). The objective of this study was to investigate the relationship between PNI and composite adverse prognostic outcomes in a cohort of hospitalized COVID-19 patients during the Omicron wave.

## Materials and methods

### Patient selection

**Fig. 1:**
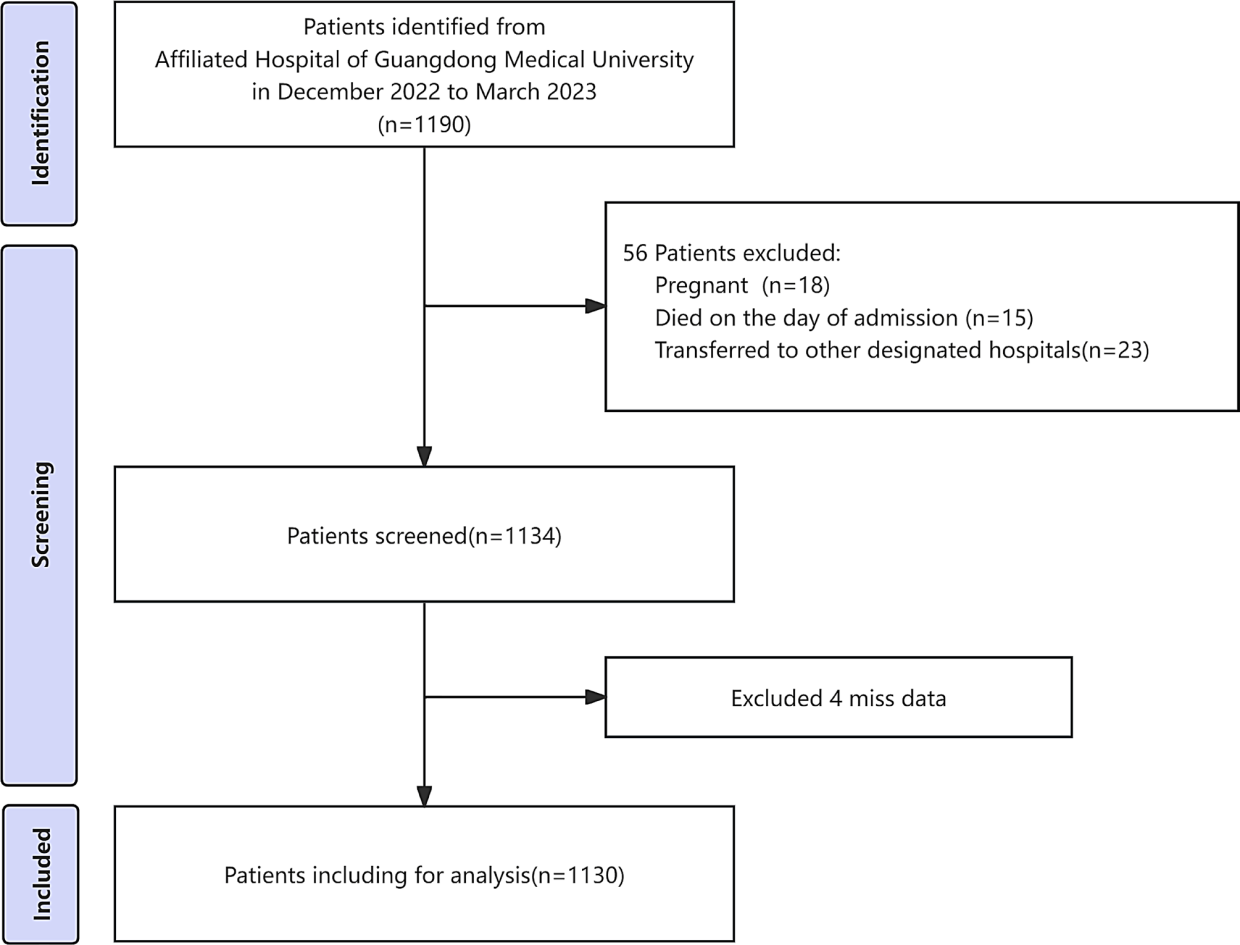
Participant selection process flowchart

Our study enrolled 1190 COVID-19 patients who were admitted to the Affiliated Hospital of Guangdong Medical University from December 2022 to March 2023. Inclusion criteria were as follows: (1) patients afflicted with COVID-19 are diagnosed in accordance with 10th edition of the COVID-19 Diagnosis and Treatment Plan issued by the National Health Commission of China [18]; (2) completed blood tests after being admitted to the hospital. Exclusion criteria include: (1) patients who under 18 years old or over 85 years old; (2) patients who were pregnant; (3) patients who died on the day of admission; (4) patients who were transferred to other designated hospitals during hospitalization; (5) patients with incomplete baseline data. Lastly, 1130 patients were included in the final analysis (Fig. 1). Given its retrospective study design, informed consent was waived. All procedures in this study were performed in accordance with the Declaration of Helsinki and were approved by the Medical Ethics Committee of the Affiliated Hospital of Guangdong Medical University (Ethics Committee number ChiMCTR2000003220, Approval number PJ2020-026).

### Data sources

#### Assessment of covariates

The entirety of the data employed in this investigation was derived from the hospital’s electronic informatics system. The demographic data (age and sex), clinical type (non-severe cases [mild or moderate] or severe cases [severe or very severe cases]), death during hospitalization, hospitalization (ICU, critical care unit, general ward), treatment (antiviral therapy [Paxlovid], hormones therapy, non-mechanical ventilation [NMV], invasive mechanical ventilation [IMV], or non-invasive mechanical ventilation [NIMV]), comorbidities (coronary artery disease, COPD, diabetes, cerebral infarction, tumor, chronic kidney disease), imaging features (consolidation or ground-glass opacity), and laboratory parameters (white blood cell [WBC], haemoglobin [HGB], LY, C-reactive protein [CRP], procalcitonin [PCT], blood urea nitrogen [BUN], glomerular filtration rate [eGFR], serum creatinine [Scr], cystatin C [CysC], ALB, D-dimer [DD.i], activated partial thromboplastin time [APTT], and prothrombin time [PT]) were collected from hospital databases. The study identified underlying comorbidities such as diabetes, chronic kidney disease, coronary heart disease, stroke, chronic obstructive pulmonary disease (COPD), and tumors as potential risk factors for hospitalization. In addition, the Charlson Comorbidity Index (CCI) [19] which was calculated from published methods, was calculated as a potential risk factor.

#### Assessment of the PNI

The PNI is calculated by the patient’s LY count and ALB level. The specific calculation formula was as follows: PNI = 5 × LY count (10^9/L) + ALB level (g/L) [20]. The LY count refers to the number of LY obtained on blood tests, and the unit is 10^9/L. The ALB level is the concentration of plasma albumin obtained in a blood test in g/L. Patients were divided into four groups (Q1–Q4) according to the quartile of the PNI, and group Q1 was used as the control group.

#### Ascertainment of adverse prognostic outcomes

An adverse prognostic outcome happened when one of the following conditions occurred: death, intubation, mechanical ventilation, or admission to the ICU for acute exacerbation. Mechanical ventilation and intubation are common emergency treatments for critical COVID-19 patients. Death is the final outcome of patients in critical condition. Most critical COVID-19 patients will be arranged to stay in ICU and critical care units, although it may be occasionally affected by hospital protocols and bed availability. Therefore, we used the composite outcomes for COVID-19 patients to assess their adverse prognostic outcomes. All prognostic outcomes were derived from medical records.

### Statistical analysis

We used multiple imputations (MIs), based on five replications and a chained equation approach method in the R MI procedure [21, 22]. Notably, the majority of patients (27.6%) lacked information on PCT, 25.2% on CRP, 17.7% on DD.i, 11.1% on APTT, and 11.0% on PT. To ensure the stability of the statistical model, patients with outlying values for the PNI were excluded via the interquartile range (IQR) method. The final analysis included 1117 patients.

Firstly, the baseline data were statistically described according to the PNI quartile grouping, in which continuous variables are represented as the mean ± standard deviation (SD), and categorical variables are expressed as numbers and percentages. One-way analysis of variance (ANOVA), the Kruskal‒Wallis test, and the chi‒square test were used to assess the differences between these groups.

Secondly, univariate logistic regression was employed to assess the relationship between various factors and adverse prognosis outcomes. Thirdly, multivariate logistic regression was used to assess the association between PNI and adverse prognosis outcomes and each component, when PNI was considered as both a continuous variable and a categorical variable. Variables exhibiting statistically significant associations in the univariate analysis were included in the following three models. Model 1 was left unadjusted for any variables; Model 2 was adjusted for age, sex, and the CCI; and Model 3 was adjustment for age, sex, CCI, nasal oxygen, antiviral therapy, hormone therapy, SPO2, Lung CT, chronic kidney disease, COPD, WBC, HGB, CRP, PCT, Scr, CysC, eGFR, BUN, DD.i, PT, APTT. Correlation analysis among the components was conducted employing an Upset Plot and a Phi Coefficient Matrix. Furthermore, we conducted a sensitivity analysis on the complete cases with the imputed dataset. The odds ratio (OR) and 95% confidence interval (95%CI) were calculated accordingly. Additionally, P for trend analysis was conducted to clarify overall differences when the PNI was treated as a categorical variable. The predictive accuracy of these risk factors was assessed using Receiver Operating Characteristic (ROC) curves. Fourthly, subgroup analyses were performed to compare the relationships between the PNI and adverse prognosis outcomes among different characteristic populations, highlighting any differences through interaction effects. Fifthly, RCS curves were fitted to assess the risk of adverse prognosis outcomes on the basis of the PNI. All analyses were performed utilizing SPSS and R. A *p*-value < 0.05 was considered statistically significant.

## Results

### Baseline characteristics

A total of 1117 patients were included in the final analysis, with a (mean ± SD) age of (70.94 ± 14.01) years in the cohort and 66.1% of participants are male. The total number of patients with adverse prognostic outcomes was 415 (37.2%). Table 1 shows the baseline demographic and biochemical characteristics compared with the PNI quartiles. There were statistically significant differences in the PNI quartiles, including age, sex, CCI, death, hospitalization status, intubation, mechanical ventilation, oxygen, antiviral therapy, hormone therapy, nasal oxygen, lung CT, clinical type, chronic kidney disease, COPD, cerebral infarction, WBC, LY, HGB, PCT, CRP, DD.i, BUN, Scr, CysC, ALB, eGFR, APTT, PT, and hospital outcome. Moreover, ALB and the eGFR increased with increasing PNI, but the WBC, PCT, CRP, BUN, Scr, CysC, APTT and PT decreased. Among the PNI quartiles, the risk of adverse prognostic outcomes was greatest in Q1 (174 [62.1%], versus 119 [43%], versus 78 [27.9%], versus 44 [15.7%], *P* < 0.001). The patients with high PNI values (Q4) were the youngest, and the LY count, ALB level, and HGB level were greater than those in the lower PNI groups (Q1, Q2, and Q3) (*P* < 0.001).

**Table 1:** Baseline characteristics according to PNI quartiles

	Overall	Q1	Q2	Q3	Q4	*P* value
n	1117	280	277	280	280	
PNI (mean ± SD)	40.01 ± 7.17	30.82 ± 3.35	37.61 ± 1.42	42.42 ± 1.46	49.15 ± 3.15	< 0.001
Sex (%)						< 0.001
Male	738 (66.1)	206 (73.6)	193 (69.7)	186 (66.4)	153 (54.6)	
Female	379 (33.9)	74 (26.4)	84 (30.3)	94 (33.6)	127 (45.4)	
Age (mean ± SD)	70.94 ± 14.01	74.42 ± 12.67	72.53 ± 13.94	71.10 ± 13.59	65.73 ± 14.35	< 0.001
CCI (mean ± SD)	5.00 ± 2.16	5.55 ± 1.96	5.19 ± 2.14	4.82 ± 2.10	4.44 ± 2.30	< 0.001
Death (%)						< 0.001
No	921 (82.5)	170 (60.7)	230 (83.0)	257 (91.8)	264 (94.3)	
Yes	196 (17.5)	110 (39.3)	47 (17.0)	23 (8.2)	16 (5.7)	
Hospitalization status						< 0.001
General wards	749 (67.1)	121 (43.2)	167 (60.3)	214 (76.4)	247 (88.2)	
Intensive care unit	82 (7.3)	38 (13.6)	27 (9.7)	11 (3.9)	6 (2.1)	
Respiratory Intensive Care Unit	27 (2.4)	10 (3.6)	10 (3.6)	5 (1.8)	2 (0.7)	
Geriatric Intensive Care Unit	23 (2.1)	10 (3.6)	6 (2.2)	6 (2.1)	1 (0.4)	
Emergency Department Intensive Care Unit	35 (3.1)	21 (7.5)	5 (1.8)	4 (1.4)	5 (1.8)	
Psychiatric Intensive Care Unit	10 (0.9)	3 (1.1)	3 (1.1)	3 (1.1)	1 (0.4)	
Intubate (%)						< 0.001
No	967 (86.6)	206 (73.6)	233 (84.1)	262 (93.6)	266 (95.0)	
Yes	150 (13.4)	74 (26.4)	44 (15.9)	18 (6.4)	14 (5.0)	
Mechanical ventilation (%)						< 0.001
No	932 (83.4)	190 (67.9)	219 (79.1)	260 (92.9)	263 (93.9)	
Noninvasive	65 (5.8)	32 (11.4)	22 (7.9)	6 (2.1)	5 (1.8)	
Invasive	120 (10.7)	58 (20.7)	36 (13.0)	14 (5.0)	12 (4.3)	
Nasal oxygen (%)						< 0.001
No	320 (28.6)	65 (23.2)	67 (24.2)	81 (28.9)	107 (38.2)	
Yes	797 (71.4)	215 (76.8)	210 (75.8)	199 (71.1)	173 (61.8)	
Antiviral therapy (%)						< 0.001
No	808 (72.3)	179 (63.9)	195 (70.4)	203 (72.5)	231 (82.5)	
Yes	309 (27.7)	101 (36.1)	82 (29.6)	77 (27.5)	49 (17.5)	
Hormone therapy (%)						< 0.001
No	560 (50.1)	104 (37.1)	123 (44.4)	143 (51.1)	190 (67.9)	
Yes	557 (49.9)	176 (62.9)	154 (55.6)	137 (48.9)	90 (32.1)	
SPO2 (mean ± SD)	92.58 ± 9.41	88.59 ± 12.08	91.71 ± 10.23	94.31 ± 6.67	95.71 ± 5.55	< 0.001
Lung CT (%)						< 0.001
No	442 (39.6)	86 (30.7)	92 (33.2)	114 (40.7)	150 (53.6)	
Yes	675 (60.4)	194 (69.3)	185 (66.8)	166 (59.3)	130 (46.4)	
Clinical types(%)						< 0.001
Mild	356 (31.9)	47 (16.8)	71 (25.6)	97 (34.6)	141 (50.4)	
Common	326 (29.2)	52 (18.6)	80 (28.9)	102 (36.4)	92 (32.9)	
Serious	271 (24.3)	95 (33.9)	77 (27.8)	64 (22.9)	35 (12.5)	
Critical	164 (14.7)	86 (30.7)	49 (17.7)	17 (6.1)	12 (4.3)	
Diabetes (%)						0.375
No	843 (75.5)	210 (75.0)	201 (72.6)	211 (75.4)	221 (78.9)	
Yes	274 (24.5)	70 (25.0)	76 (27.4)	69 (24.6)	59 (21.1)	
Chronic kidney disease (%)						< 0.001
No	894 (80.0)	187 (66.8)	206 (74.4)	245 (87.5)	256 (91.4)	
Yes	223 (20.0)	93 (33.2)	71 (25.6)	35 (12.5)	24 (8.6)	
Coronary artery disease (%)						0.309
No	895 (80.1)	233 (83.2)	213 (76.9)	226 (80.7)	223 (79.6)	
Yes	222 (19.9)	47 (16.8)	64 (23.1)	54 (19.3)	57 (20.4)	
COPD (%)						0.003
No	985 (88.2)	249 (88.9)	230 (83.0)	245 (87.5)	261 (93.2)	
Yes	132 (11.8)	31 (11.1)	47 (17.0)	35 (12.5)	19 (6.8)	
Cerebral infarction(%)						0.014
No	885 (79.2)	215 (76.8)	209 (75.5)	221 (78.9)	240 (85.7)	
Yes	232 (20.8)	65 (23.2)	68 (24.5)	59 (21.1)	40 (14.3)	
Tumor (%)						0.789
No	1064 (95.3)	269 (96.1)	262 (94.6)	268 (95.7)	265 (94.6)	
Yes	53 (4.7)	11 (3.9)	15 (5.4)	12 (4.3)	15 (5.4)	
WBC (mean ± SD)	7.87 ± 4.70	8.77 ± 5.39	8.33 ± 4.71	7.44 ± 4.75	6.96 ± 3.55	< 0.001
LY (mean ± SD)	1.06 ± 0.63	0.66 ± 0.41	0.86 ± 0.42	1.09 ± 0.51	1.64 ± 0.66	< 0.001
HGB (mean ± SD)	117.26 ± 26.10	105.53 ± 27.04	113.71 ± 24.99	121.70 ± 23.49	128.06 ± 23.15	< 0.001
PCT (mean ± SD)	2.00 ± 7.40	3.58 ± 9.63	2.39 ± 9.34	1.32 ± 5.46	0.73 ± 2.36	< 0.001
CRP (mean ± SD)	55.34 ± 65.94	84.94 ± 82.34	62.11 ± 64.93	44.08 ± 52.04	30.29 ± 45.29	< 0.001
DD.i (mean ± SD)	3.31 ± 8.83	5.36 ± 11.41	3.97 ± 11.94	1.97 ± 3.95	1.97 ± 4.10	< 0.001
BUN (mean ± SD)	8.83 ± 8.32	12.32 ± 10.56	9.54 ± 8.28	7:16 ± 6:45	6.30 ± 5.86	< 0.001
Scr (mean ± SD)	149.22 ± 215.38	189.07 ± 247.51	176.80 ± 249.25	122.57 ± 193.94	108.73 ± 143.24	< 0.001
CysC (mean ± SD)	1.44 ± 1.09	1.77 ± 1.24	1.63 ± 1.27	1.24 ± 0.84	1.11 ± 0.81	< 0.001
ALB (mean ± SD)	34.69 ± 5.79	27.52 ± 3.50	33.28 ± 2.45	36.98 ± 2.78	40.95 ± 3.16	< 0.001
eGFR (mean ± SD)	67.66 ± 29.44	59.18 ± 32.17	62.33 ± 32.41	72.86 ± 25.88	76.23 ± 22.87	< 0.001
APTT (mean ± SD)	37.98 ± 9.52	39.85 ± 8.35	38.31 ± 9.88	37.85 ± 11.41	35.92 ± 7.63	< 0.001
PT (mean ± SD)	13.98 ± 2.68	14.93 ± 3.06	14.00 ± 2.87	13.71 ± 2.14	13.28 ± 2.26	< 0.001
Hospital outcome (%)						< 0.001
Non-severe	702 (62.8)	106 (37.9)	158 (57.0)	202 (72.1)	236 (84.3)	
Severe	415 (37.2)	174 (62.1)	119 (43.0)	78 (27.9)	44 (15.7)	

Notes: PNI: Prognostic Nutritional Index; CCI: Charlson Comorbidity Index; COPD: Chronic obstructive pulmonary disease; WBC: White blood cell; LY: Lymphocyte absolute value; HGB: Hemoglobin; PCT: Procalcitonin; CRP: C-reactive protein; DD.i: D dimer; BUN: Blood urea nitrogen; Scr: Creatinine; CysC: cystatin C; eGFR: Estimated glomerular filtration rate; ALB: Albumin; APTT: Activated partial thromboplastin time; PT: Plasma prothrombin time

### Relationships between baseline variables and adverse prognostic outcomes

Table 2 shows a univariate logistic regression model between baseline variables and adverse prognostic outcomes. Univariate analysis revealed that age, nasal oxygen, antiviral therapy, hormone therapy, CCI, lung CT, clinical type, chronic kidney disease, COPD, cerebral infarction, WBC count, PCT, DD.i., BUN, CysC, APTT and PT were positively associated with adverse prognostic outcomes. Female, SPO2, LY, HGB, eGFR and ALB were inversely associated with adverse prognostic outcomes.

**Table 2:** Relationship between baseline variables and adverse prognostic outcomes

Variable	Statistics	OR(95%CI)	*P* value
PNI (mean ± SD)	40.01 ± 7.17	0.89(0.87–0.91)	< 0.001
Sex (%)			0.03
Male	738 (66.1)	Ref	
Female	379 (33.9)	0.75(0.58–0.97)	
Age (mean ± SD)	70.94 ± 14.01	1.02(1.01–1.03)	< 0.001
CCI (mean ± SD)	5.00 ± 2.16	1.19(1.13–1.26)	< 0.001
Nasal oxygen (%)			0.01
No	320 (28.6)	Ref	
Yes	797 (71.4)	1.47(1.11–1.93)	
Antiviral therapy (%)			< 0.001
No	808 (72.3)	Ref	
Yes	309 (27.7)	1.79(1.37–2.34)	
Hormone therapy (%)			< 0.001
No	560 (50.1)	Ref	
Yes	557 (49.9)	2.83(2.2–3.64)	
SPO2 (mean ± SD)	92.58 ± 9.41	0.79(0.77–0.82)	< 0.001
Lung CT (%)			< 0.001
No	442 (39.6)	Ref	
Yes	675 (60.4)	2.23(1.72–2.9)	
Clinical types(%)			
Mild	356 (31.9)	Ref	
Common	326 (29.2)	1.44(0.96–2.16)	0.08
Serious	271 (24.3)	6.44(4.4–9.45)	< 0.001
Critical	164 (14.7)	∞	0.97
Diabetes (%)			0.55
No	843 (75.5)	Ref	
Yes	274 (24.5)	1.09(0.82–1.44)	
Chronic kidney disease (%)			< 0.001
No	894 (80.0)	Ref	
Yes	223 (20.0)	2.31(1.72–3.11)	
Coronary artery disease (%)			0.31
No	895 (80.1)	Ref	
Yes	222 (19.9)	1.17(0.86–1.58)	
COPD (%)			< 0.001
No	985 (88.2)	Ref	
Yes	132 (11.8)	1.89(1.31–2.72)	
Cerebral infarction(%)			0.07
No	885 (79.2)	Ref	
Yes	232 (20.8)	1.31(0.98–1.76)	
Tumor (%)			0.7
No	1064 (95.3)	Ref	
Yes	53 (4.7)	1.12(0.63–1.96)	
WBC (mean ± SD)	7.87 ± 4.70	1.16(1.13–1.2)	< 0.001
LY (mean ± SD)	1.06 ± 0.63	0.42(0.33–0.53)	< 0.001
HGB (mean ± SD)	117.26 ± 26.10	0.99(0.98–0.99)	< 0.001
PCT (mean ± SD)	2.00 ± 7.40	1.1(1.06–1.14)	< 0.001
CRP (mean ± SD)	55.34 ± 65.94	1.01(1.01–1.01)	< 0.001
DD.i (mean ± SD)	3.31 ± 8.83	1.1(1.07–1.14)	< 0.001
BUN (mean ± SD)	8.83 ± 8.32	1.05(1.04–1.07)	< 0.001
Scr (mean ± SD)	149.22 ± 215.38	1(1.00–1.00)	0.09
CysC (mean ± SD)	1.44 ± 1.09	1.19(1.07–1.33)	< 0.001
ALB (mean ± SD)	34.69 ± 5.79	0.88(0.86–0.91)	< 0.001
eGFR (mean ± SD)	67.66 ± 29.44	0.99(0.99-1)	< 0.001
APTT (mean ± SD)	37.98 ± 9.52	1.04(1.02–1.06)	< 0.001
PT (mean ± SD)	13.98 ± 2.68	1.3(1.2–1.39)	< 0.001

Notes: PNI: Prognostic Nutritional Indexes; CCI: Charlson Comorbidity Index; COPD: Chronic obstructive pulmonary disease; WBC: White blood cell; LY: Lymphocyte absolute value; HGB: Hemoglobin; PCT: Procalcitonin; CRP: C-reactive protein; DD.i: D dimer; BUN: Blood urea nitrogen; Scr: Creatinine; CysC: cystatin C; eGFR: Estimated glomerular filtration rate; ALB: Albumin; APTT: Activated partial thromboplastin time; PT: Plasma prothrombin time

### Risk correlation between the baseline PNI and adverse prognostic outcomes

**Fig. 2:**
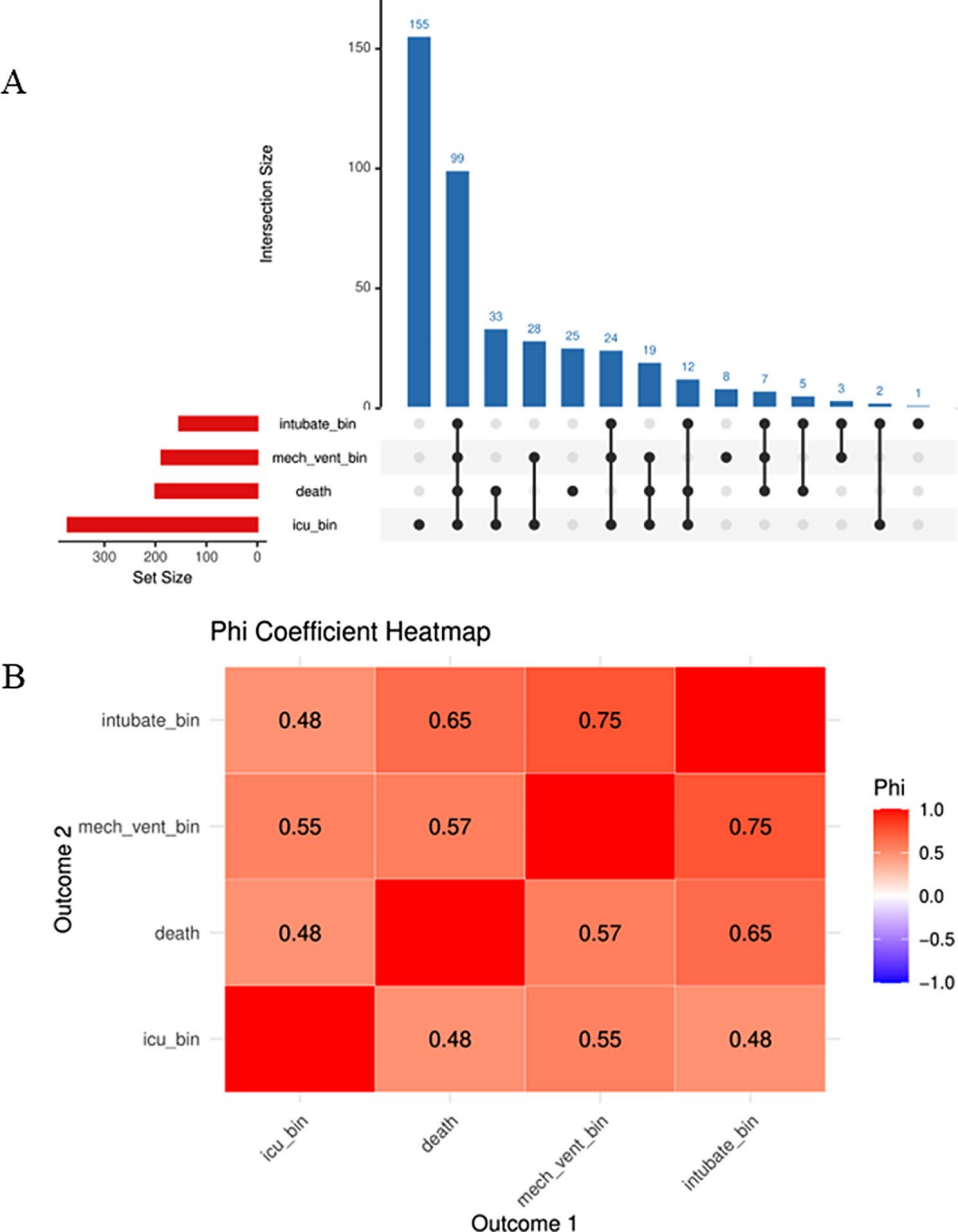
The relationship between the outcomes of each component. (**A**) Clinical Outcomes Intersection Map; (**B**) Interrelationship of Binary Clinical Outcomes (Phi Coefficient Matrix)

As shown in Table 3, the results of multivariate logistic regression models evaluated the association between PNI and the incidence of adverse prognostic outcomes. The OR for adverse prognostic outcomes significantly decreased with increasing PNI values and increasing PNI quartiles in the unadjusted model. The incidence of adverse prognostic outcomes per unit increase in the PNI was reduced by 11% (OR = 0.89, 95% CI: 0.87–0.91; *P* < 0.001). When the data were stratified into quartiles, the risk of adverse prognostic outcomes was significantly lower in Q4 than in Q1 (OR = 0.11, 95% CI: 0.08–0.17; *P* < 0.001). After adjusting for demographic variables (age and sex and the CCI), the association between PNI and the incidence of adverse prognostic outcomes remained resemble. The relationship between the PNI and the incidence of adverse prognostic outcomes remained unaffected after further adjustment for age, sex, CCI, and significant variables. The correlation analysis of the components is presented in Fig. 2 which demonstrated a robust positive correlation. The risk of adverse prognostic outcomes for the Q1 group is apparently elevated compared to the Q3 and Q4 groups in each component analysis (Supplement Table 1). The sensitivity analysis of complete cases revealed consistent the results (Supplement Table 2 and Supplement Table 3).

**Table 3:** Risk correlation between baseline PNI and adverse prognostic outcomes

	Model 1 Odds ratio (95% CI)	*P* value	Model 2 Odds ratio (95% CI)	*P* value	Model 3 Odds ratio (95% CI)	*P* value
PNI	0.89 (0.87 ,0.91)	< 0.001	0.90 (0.88 ,0.92)	< 0.001	0.95 (0.93 ,0.97)	0.001
Q1	Ref		Ref		Ref	
Q2	0.46 (0.33 ,0.64)	< 0.001	0.48 (0.34 ,0.67)	< 0.001	0.76 (0.51 ,1.13)	0.169
Q3	0.24 (0.16 ,0.33)	< 0.001	0.26 (0.18 ,0.36)	< 0.001	0.52 (0.34 ,0.80)	0.003
Q4	0.11 (0.08 ,0.17)	< 0.001	0.13 (0.09 ,0.20)	< 0.001	0.37 (0.22 ,0.60)	< 0.001
P for trend	< 0.001		< 0.001		0.004	

Model 1:Unadjusted

Model 2:Age, sex, CCI

Model 3: Age, sex, CCI, nasal oxygen, antiviral therapy, hormone therapy, SPO2, Lung CT, chronic kidney disease, COPD, WBC, HGB, CRP, PCT, Scr, CysC, eGFR, BUN, DD.i, PT, APTT

### Predicted value of PNI for adverse prognostic outcomes

**Fig. 3:**
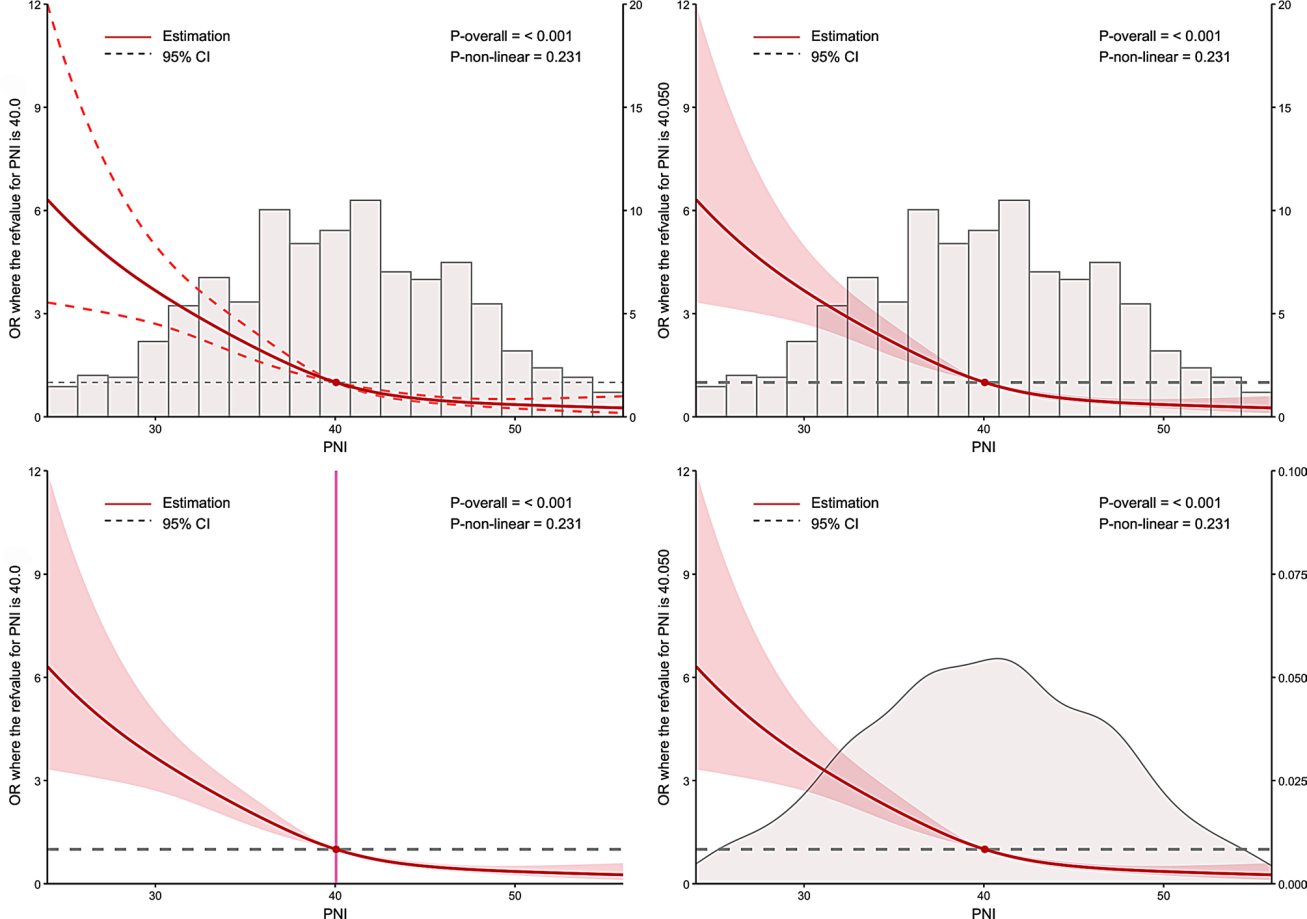
Restricted cubic spline analysis of PNI and outcome risk

**Fig. 4:**
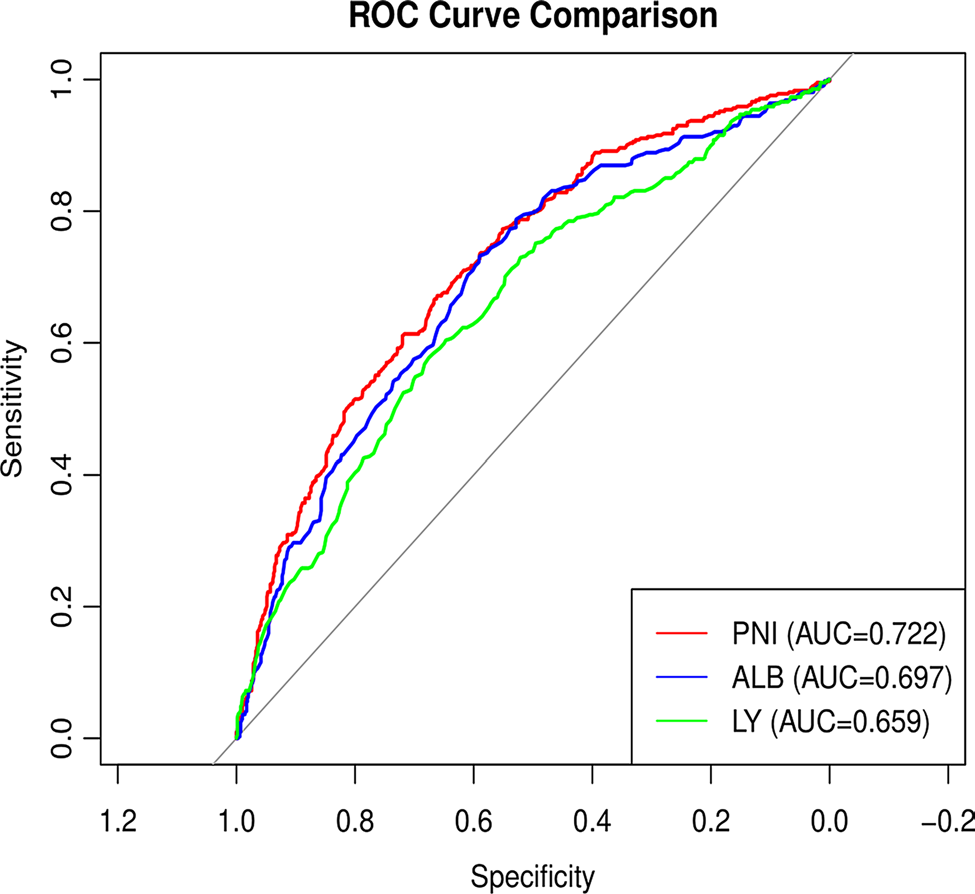
ROC curve for adverse prognostic outcomes

The relationship was observed between PNI and adverse prognostic outcomes, as illustrated in the RCS curve analysis (Fig. 3). As PNI decreased below the reference value of 40, the OR for adverse prognostic outcomes increased substantially, supporting the role of low PNI as an independent predictor of adverse prognostic outcomes. ROC curve analysis was employed to examine the relationship between the PNI and adverse prognostic outcomes, with the associations of ALB and LY counts with adverse prognostic outcomes also being evaluated. It demonstrated that PNI exhibited the highest discriminatory ability for the outcome of interest, with an area under the curve (AUC) of 0.722 (95%CI: 0.691–0.752), with a cutoff of 39.3, yielding a sensitivity of 0.671 and a specificity of 0.661. ALB also showed moderate predictive performance with an AUC of 0.697, while LY had the lowest predictive value (AUC = 0.659). For further details, refer to Table 4; Fig. 4.

**Table 4:** Prediction value for adverse prognostic outcomes

Marker	AUC(95%CI)	Cutoff	Sensitivity	Specificity	Accuracy
PNI	0.722(0.691–0.752)	39.3	0.671	0.661	0.665
ALB	0.697(0.665–0.729)	35.45	0.732	0.590	0.643
LY	0.659(0.626–0.692)	0.815	0.577	0.676	0.639

Notes: PNI: Prognostic Nutritional Indexes; ALB: Albumin; LY: Lymphocyte absolute value

### Subgroup analysis

We performed a subgroup analysis to determine the concordance between the PNI and adverse prognostic outcomes in COVID-19 patients (Table 5), considering the effects of sex (Female or Male), age (≤ 75 or > 75 years), oxygen (Yes or No), antiviral therapy (Yes or No), hormone therapy (Yes or No), and pulmonary nodules in lung CT (Yes or No). In the stratified analysis, the associations between these factors were similar across all strata (*P* > 0.05).

**Table 5:** Correlation between PNI and adverse prognostic outcomes in subgroups

Variable		Count	Percent	OR(95%CI)	*P* value	*P* for interaction
Sex						0.099
	Male	738	66.1	0.72(0.38–1.33)	0.294	
	Female	379	33.9	0.84(0.33–2.16)	0.718	
Age						0.162
	<75	619	55.4	0.69(0.32–1.49)	0.339	
	≥75	498	44.6	0.84(0.41–1.7)	0.623	
Nasal oxygen						0.977
	No	320	28.6	1.19(0.11–13.16)	0.883	
	Yes	797	71.4	0.81(0.48–1.37)	0.426	
Antiviral therapy						0.301
	No	808	72.3	0.72(0.38–1.33)	0.29	
	Yes	309	27.7	1.26(0.5–3.23)	0.628	
Hormone therapy						0.073
	No	560	50.1	0.44(0.19–1.01)	0.054	
	Yes	557	49.9	1.15(0.59–2.24)	0.677	
Lung CT						0.73
	No	442	39.6	0.76(0.25–2.28)	0.621	
	Yes	675	60.4	0.92(0.51–1.67)	0.783	

Adjust: Sex, age, CCI, intubate, mechanical ventilation, SPO2, nasal oxygen, antiviral therapy, hormone therapy, WBC, HGB, PCT, CRP, DD.i, BUN, Scr, CysC, eGFR, APTT, PT

## Discussion

This retrospective study suggests that low PNI is an independent predictor of adverse prognosis outcomes in COVID-19 patients. Previous studies have demonstrated that the PNI is an important predictive factor for the prognosis of hospitalized COPD patients [23], as well as an independent predictor for adverse prognostic outcomes in patients with severe traumatic brain injury (STBI) [24]. Furthermore, the PNI has been shown to effectively predict adverse prognostic outcomes in patients for elderly patients with nonmetastatic nasopharyngeal carcinoma [25], and patients with metabolic syndrome complicated by heart failure [26]. The study found that lower PNI is important in predicting the risk of death on admission in COVID-19 patients, which confirmed by Wei et al. [15]. In our study, the cutoff value of the PNI for predicting adverse prognostic outcomes in COVID-19 patients was 39.3, whereas Wang et al. [27] identified that the optimal PNI cutoff value was 38.04.The discrepancy between the two values may stem from multiple factors, including population differences and the Omicron variant. Considering the findings of this study, clinicians can perform nutritional assessments to stratify patients and subsequently implement targeted interventions for those with low PNI, which may help reduce the risk of clinical deterioration in hospitalized COVID-19 patients. However, it is extremely complex that the low PNI may be caused by impaired immune function due to malnutrition, while severe illness can exacerbate nutritional deficiencies. Clinicians must remain cautious about this harmful cycle.

The PNI is calculated on the basis of the total LY count and serum ALB concentration, which can be easily obtained through routine peripheral blood tests [28]. A low PNI, which reflects both LY and hypoalbuminemia, may indicate impaired immune function and systemic catabolic or inflammatory status. The pathophysiological mechanisms underlying the association between low PNI and adverse outcomes in COVID-19 are likely multifactorial. Lymphopenia, one component of PNI, reflects immune exhaustion and impaired host defence, both of which are common features of severe COVID-19. A decrease in LY count, particularly in CD4⁺ and CD8⁺ T-cell subsets, compromises the ability to clear viral infection and leads to dysregulated immune responses, resulting in persistent viral replication, cytokine overproduction, and tissue damage [14, 29, 30]. Meanwhile, hypoalbuminemia, the other component of PNI, is a well-recognized marker of systemic inflammation and poor nutritional status. During severe SARS-CoV-2 infection, inflammatory cytokines such as interleukin-6 (IL-6) and tumor necrosis factor-α (TNF-α) suppress hepatic albumin synthesis and increase vascular permeability, promoting extravascular albumin leakage [31]. Low serum ALB levels therefore reflect both an ongoing inflammatory process and a catabolic state, which contribute to organ dysfunction and worse clinical outcomes in COVID-19 patients [9, 29]. Collectively, a low PNI integrates the deleterious effects of immune suppression and systemic inflammation, representing a state of immune–nutritional depletion. This dual impairment weakens the host’s ability to control viral infection and recover from tissue injury, thereby predisposing patients in COVID-19 to severe disease and mortality.

The PNI is a comprehensive indicator that evaluates protein levels, nutritional status, and immune function, commonly employed to assess the nutritional condition of elderly individuals and those with chronic illnesses. In contrast to BMI, which primarily assesses physique through weight and height, PNI effectively reflects the body’s immune function and nutritional reserves. Therefore, PNI provides greater sensitivity in evaluating an individual’s nutritional risk, prognosis, and recovery potential. Therefore, PNI is particularly valuable for its ability to detect potential malnutrition and immunocompromised early in the elderly and those with serious medical conditions.

A study by Wang et al. [32] showed that COVID-19 patients often experience fever, cough, difficulty breathing, and loss of taste and smell, leading to malnutrition. Inflammation, limited physical activity, disruption of metabolic balance and synthesis, and endocrine dysfunction are also contributing to malnutrition in COVID patients [6, 7, 33]. Concurrently, malnutrition impairs the function of the immune system and increases the risk of contracting COVID-19, leading to an increased risk of adverse prognosis outcomes [34].

In this study, we conducted research on Asian populations primarily infected with the Omicron wave during 2022–2023. Compared with other studies [14, 15], larger cohorts and detailed clinical data were analysed in our study during the Omicron wave. Multivariable adjustment and RCS analysis have been used to enhance the evidence. The PNI value was 40 as the reference in our research, which is similar to the value (PNI = 40.72) reported in Wang et al.’s study [27]. Consistent with previous studies [16, 17], our findings indicate that PNI is a predictive factor in the prognosis of COVID-19. Furthermore, this study may be influenced by potential confounding factors due to the limitations of retrospective observational research. To minimize the impact of confounding factors, we employed rigorous statistical adjustment methods and examined multicollinearity. Additionally, we examined the robustness of the results through subgroup analysis. The predictive capability of PNI is consistent across different subgroups such as gender and age.

In this cohort of 1,117 patients, the average age was approximately 70 years, with male patients accounting for 66.1%. The prognostic value of PNI as a predictor in hospitalized elderly patients with COVID-19 was validated [14, 16, 17]. However, our study has several limitations that may affect the reliability of the conclusions, which should be considered when interpreting the findings. Firstly, lots of elderly individuals and those unvaccinated were severely affected during the COVID-19 pandemic driven by the Omicron wave in 2022–2023. Regrettably, vaccination status could not be collected in this study. Consequently, its applicability to other COVID-19 variants, unvaccinated and non-hospitalized patients remains to be substantiated. Secondly, data were missing for certain covariates (such as PCT, CRP, DD.i, APTT, PT, etc.), with a missing data rate exceeding 10%. We address the issue of missing data through multiple imputations, and the results remain robust. Thirdly, our research observed the period from admission to discharge of COVID-19 patients. We were unable to follow up or track the changes in the COVID-19 patients’ conditions after discharge, which may lead to bias. Fifthly, serial monitoring of changes in PNI during treatment may be associated with adverse prognostic outcomes in COVID-19 patients. Unfortunately, the dynamic changes in PNI were not recorded in our research. Finally, although we adjusted for multiple confounding factors in our analysis, it is still possible that residual confounding factors (including socioeconomic status and smoking history) are present and cannot be completely ruled out. Moreover, other unmeasured confounders such as vaccination status and nutritional support should not be overlooked. This study is limited by its retrospective, single-center design, which may introduce selection bias and unmeasured confounding. Therefore, our findings should be interpreted with caution and confirmed in larger, prospective, multicenter studies. Future studies should further investigate the serial monitoring of changes in PNI during disease treatment, which may hold greater significance for reducing the risk of adverse prognostic outcomes.

## Conclusions

In conclusion, our study revealed a significant association between the low PNI and adverse prognosis outcomes in COVID-19 patients. PNI was an independent predictor of the happened of intubation, mechanical ventilation, death, or admission to the ICU in COVID-19 patients.

## Supplementary Information

Below is the link to the electronic supplementary material.

Supplementary Material 1

## Data Availability

The data that support the findings of this study are not openly available due to reasons of sensitivity and are available from the corresponding author upon reasonable request. Data are located in controlled access data storage at the Affiliated Hospital of Guangdong Medical University.

## Abbreviations

PNI: Prognostic Nutritional Index
SARS-CoV-2: Severe Acute Respiratory Syndrome Coronavirus 2
BMI: Body Mass Index
CI: Confidence Intervals
OR: Odds Ratios
COPD: Chronic Obstructive Pulmonary Disease
WBC: White Blood Cell
HGB: Haemoglobin
CRP: C-reactive Protein
PCT: Procalcitonin
BUN: Blood Urea Nitrogen
eGFR: Estimated Glomerular Filtration Rate
Scr: Serum Creatinine
CysC: Cystatin C
ALB: Albumin
DD.i: D-dimer
APTT: Activated Partial Thromboplastin Time
PT: Prothrombin Time
CCI: Charlson Comorbidity Index
IQR: Interquartile Range
STBI: Severe Traumatic Brain Injury
APC: Antigen-presenting Cell
