# Supplementary material for: Prognostic nutritional index is a predictor of adverse outcomes in hospitalized COVID-19 patients: a single-center, retrospective cohort study
Source: BMC Infect Dis. 2025 Dec 1;26:15. doi: 10.1186/s12879-025-12244-z (PMC12781248; doi:10.1186/s12879-025-12244-z)
Supplement: Supplementary file 1 — Supplementary Material 1 [file 12879_2025_12244_MOESM1_ESM.docx]

Supplement Table 1 Risk correlation between baseline PNI and adverse prognostic outcomes for each component.

|  | Model 1 Odds ratio (95% CI) | *P* value | Model 2 Odds ratio (95% CI) | *P* value | Model 3 Odds ratio (95% CI) | *P* value |
| --- | --- | --- | --- | --- | --- | --- |
| Adverse prognostic outcomes for death | | | | | | |
| PNI | 0.87 (0.84 ,0.89) | <0.001 | 0.88 (0.85 ,0.90) | <0.001 | 0.93 (0.86 ,0.99) | 0.047 |
| Q1 | Ref |  | Ref |  | Ref |  |
| Q2 | 10.55 (5.92 ,18.77) | <0.001 | 8.72 (4.83 ,15.75) | <0.001 | 2.68 (0.89 ,8.12) | 0.079 |
| Q3 | 3.54 (1.91 ,6.54) | <0.001 | 3.02 (1.62 ,5.66 ) | <0.001 | 1.48 (0.64 ,3.47) | 0.359 |
| Q4 | 1.60 (0.81 ,3.13) | 0.174 | 1.42 (0.72 ,2.82) | 0.304 | 1.01 (0.46 ,2.21) | 0.971 |
| Adverse prognostic outcomes for intubation | | | | | | |
| PNI | 0.89 (0.87 ,0.92) | <0.001 | 0.90 (0.88 ,0.93) | <0.001 | 0.91 (0.84 ,0.99) | 0.013 |
| Q1 | Ref |  | Ref |  | Ref |  |
| Q2 | 6.50 (3.55 ,11.89) | <0.001 | 5.25 (2.83 ,9.74) | <0.001 | 2.06 (0.63 ,6.76) | 0.234 |
| Q3 | 3.46 (1.85 ,6.47) | <0.001 | 2.94 (1.55 ,5.57) | <0.001 | 0.77 (0.74 ,4.27) | 0.201 |
| Q4 | 1.29 (0.63 ,2.64) | 0.491 | 1.14 (0.55 ,2.36) | 0.724 | 0.88 (0.38 ,2.03) | 0.766 |
| Adverse prognostic outcomes for admission to the ICU | | | | | | |
| PNI | 0.88 (0.86 ,0.90) | <0.001 | 0.89 (0.87 ,0.91) | <0.001 | 0.89 (0.84 ,0.94) | <0.001 |
| Q1 | Ref |  | Ref |  | Ref |  |
| Q2 | 10.27 (6.56 ,16.07) | <0.001 | 8.63 (5.46 ,13.65) | <0.001 | 5.52 (2.25 ,13.52) | <0.001 |
| Q3 | 5.12 (3.26 ,8.04) | <0.001 | 4.51 (2.85 ,7.14) | <0.001 | 3.66 (1.89 ,7.07) | <0.001 |
| Q4 | 2.42 (1.50 ,3.91) | <0.001 | 2.21 (1.36 ,3.58) | 0.001 | 1.99 (1.11 ,3.56) | 0.021 |
|  |  |  |  |  |  |  |
| Adverse prognostic outcomes for mechanical ventilation | | | | | | |
| PNI | 0.89 (0.87 ,0.91) | <0.001 | 0.90 (0.87 ,0.92) | <0.001 | 0.89 (0.82 ,0.96) | 0.003 |
| Q1 | Ref |  | Ref |  | Ref |  |
| Q2 | 7.41 (4.18 ,13.14) | <0.001 | 6.11 (3.39 ,10.99) | <0.001 | 3.02 (0.98 ,9.32) | 0.054 |
| Q3 | 4.27 (2.36 ,7.71) | <0.001 | 3.70 (2.03 ,6.74) | <0.001 | 2.47 (1.07 , 5.70) | 0.034 |
| Q4 | 1.32 (0.67 ,2.62) | 0.421 | 1.18 (0.59 , 2.36 ) | 0.637 | 0.88 (0.39 ,1.98) | 0.761 |

Model 1:Unadjusted;

Model 2:Age, sex, CCI;

Model 3: Age, sex, CCI, nasal oxygen, antiviral therapy, hormone therapy, SPO2, Lung CT, chronic kidney disease, COPD, WBC, HGB, CRP, PCT, Scr, CysC, eGFR, BUN, DD.i, PT, APTT.

Supplement Table 2 Comparison between the imputed datasets and complete cases.

|  | Imputed datasets | Complete case | P value |
| --- | --- | --- | --- |
| n | 1117 | 558 |  |
| PNI (mean±SD) | 40.01±7.17 | 42.55±8.40 | 0.309 |
| Sex (%) |  |  | 0.937 |
| Male | 738 (66.1) | 370 (66.3) |  |
| Female | 379 (33.9) | 188 (33.7) |  |
| Age (mean±SD) | 70.94±14.01 | 71.87±13.96 | 0.193 |
| CCI (mean±SD) | 5.00±2.16 | 5.32±2.14 | 0.004 |
| Death (%) |  |  | 0.988 |
| No | 921 (82.5) | 461 (82.6) |  |
| Yes | 196 (17.5) | 97 (17.4) |  |
| Hospitalization status | |  | 0.127 |
| General wards | 749 (67.1) | 345 (61.8) |  |
| Intensive care unit | 82 (7.3) | 61 (10.9) |  |
| Respiratory Intensive Care Unit | 27 (2.4) | 13 (2.3) |  |
| Geriatric Intensive Care Unit | 23 (2.1) | 16 (2.9) |  |
| Emergency Department Intensive Care Unit | 35 (3.1) | 13 (2.3) |  |
| Psychiatric Intensive Care Unit | 10 (0.9) | 7 (1.3) |  |
| Intubate (%) | |  | 0.527 |
| No | 967 (86.6) | 476 (85.3) |  |
| Yes | 150 (13.4) | 82 (14.7) |  |
| Mechanical ventilation (%) | |  | 0.423 |
| No | 932 (83.4) | 455 (81.5) |  |
| Noninvasive | 65 (5.8) | 31 (5.6) |  |
| Invasive | 120 (10.7) | 72 (12.9) |  |
| Nasal oxygen (%) | |  | 0.023 |
| No | 320 (28.6) | 130 (23.3) |  |
| Yes | 797 (71.4) | 428 (76.7) |  |
| Antiviral therapy (%) | |  | 0.016 |
| No | 808 (72.3) | 371 (66.5) |  |
| Yes | 309 (27.7) | 187 (33.5) |  |
| Hormone therapy (%) | |  | 0.008 |
| No | 560 (50.1) | 241 (43.2) |  |
| Yes | 557 (49.9) | 317 (56.8) |  |
| SPO2 (mean±SD) | 92.58±9.41 | 92.08±8.80 | 0.288 |
| Lung CT (%) | |  | 0.002 |
| No | 442 (39.6) | 176 (31.5) |  |
| Yes | 675 (60.4) | 382 (68.5) |  |
| Clinical types(%) | |  | 0.009 |
| Mild | 356 (31.9) | 135 (24.2) |  |
| Common | 326 (29.2) | 172 (30.8) |  |
| Serious | 271 (24.3) | 163 (29.2) |  |
| Critical | 164 (14.7) | 88 (15.8) |  |
| Diabetes (%) | |  | 0.046 |
| No | 843 (75.5) | 395 (70.8) |  |
| Yes | 274 (24.5) | 163 (29.2) |  |
| Chronic kidney disease (%) | |  | 0.012 |
| No | 894 (80.0) | 416 (74.6) |  |
| Yes | 223 (20.0) | 142 (25.4) |  |
| Coronary artery disease (%) | |  | 0.423 |
| No | 895 (80.1) | 437 (78.3) |  |
| Yes | 222 (19.9) | 121 (21.7) |  |
| COPD (%) |  |  | 0.898 |
| No | 985 (88.2) | 494 (88.5) |  |
| Yes | 132 (11.8) | 64 (11.5) |  |
| Cerebral infarction(%) | |  | 0.012 |
| No | 885 (79.2) | 411 (73.7) |  |
| Yes | 232 (20.8) | 147 (26.3) |  |
| Tumor (%) | |  | 0.452 |
| No | 1064 (95.3) | 526 (94.3) |  |
| Yes | 53 (4.7) | 32 (5.7) |  |
| WBC (mean±SD) | 7.87±4.70 | 9.44±19.36 | 0.009 |
| LY (mean±SD) | 1.06±0.63 | 1.80±16.24 | 0.127 |
| HGB (mean±SD) | 117.26±26.10 | 113.19±26.44 | 0.002 |
| PCT (mean±SD) | 2.00±7.40 | 2.25±8.13 | 0.645 |
| CRP (mean±SD) | 55.34±65.94 | 63.65±70.25 | 0.027 |
| DD.i (mean±SD) | 3.31±8.83 | 3.87±10.61 | 0.195 |
| BUN (mean±SD) | 8.83±8.32 | 9.78±9.53 | 0.031 |
| Scr (mean±SD) | 149.22±215.38 | 176.48±261.57 | 0.034 |
| CysC (mean±SD) | 1.44±1.09 | 1.59±1.24 | 0.007 |
| ALB (mean±SD) | 34.69±5.79 | 33.54±5.57 | <0.001 |
| eGFR (mean±SD) | 67.66±29.44 | 64.50±31.55 | 0.043 |
| APTT (mean±SD) | 37.98±9.52 | 38.54±9.78 | 0.198 |
| PT (mean±SD) | 13.98±2.68 | 14.13±2.80 | 0.21 |

Notes: PNI: Prognostic Nutritional Index; CCI: Charlson Comorbidity Index; COPD: Chronic obstructive pulmonary disease; WBC: White blood cell; LY: Lymphocyte absolute value; HGB: Hemoglobin; PCT: Procalcitonin; CRP:C-reactive protein; DD.i: D dimer; BUN: Blood urea nitrogen; Scr: Creatinine; CysC: cystatin C ;eGFR: Estimated glomerular filtration rate; ALB: Albumin; APTT: Activated partial thromboplastin time; PT: Plasma prothrombin time.

Supplement Table 3 Risk correlation between baseline PNI and adverse prognostic outcomes for complete cases (N=558).

|  | Model 1 Odds ratio (95% CI) | *P* value | Model 2 Odds ratio (95% CI) | *P* value | Model 3 Odds ratio (95% CI) | *P* value |
| --- | --- | --- | --- | --- | --- | --- |
| PNI | 0.91 (0.88 ,0.93) | <0.001 | 0.91 (0.88 ,0.94) | <0.001 | 0.91 (0.84 ,0.99) | 0.023 |
| Q1 | Ref |  | Ref |  | Ref |  |
| Q2 | 0.63 (0.39 ,1.02) | 0.059 | 0.66 (0.40 ,1.08) | 0.099 | 0.98 (0.49 ,1.94) | 0.944 |
| Q3 | 0.41 (0.25 ,0.67) | <0.001 | 0.46 (0.28 ,0.75) | 0.002 | 0.75 (0.32 ,1.76) | 0.504 |
| Q4 | 0.20 (0.12 ,0.34) | <0.001 | 0.23 (0.13 ,0.39) | <0.001 | 0.41 (0.13 ,1.26) | 0.124 |

Model 1:Unadjusted;

Model 2:Age, sex, CCI;

Model 3: Age, sex, CCI, nasal oxygen, antiviral therapy, hormone therapy, SPO2, Lung CT, chronic kidney disease, COPD, WBC, HGB, CRP, PCT, Scr, CysC, eGFR, BUN, DD.i, PT, APTT.
